# Inbreeding depression and the probability of racing in the Thoroughbred horse

**DOI:** 10.1098/rspb.2022.0487

**Published:** 2022-06-29

**Authors:** Emmeline W. Hill, Martin A. Stoffel, Beatrice A. McGivney, David E. MacHugh, Josephine M. Pemberton

**Affiliations:** ^1^ Plusvital Ltd, The Highline, Dún Laoghaire Industrial Estate, Pottery Road, Dún Laoghaire, Co. Dublin, Ireland; ^2^ UCD School of Agriculture and Food Science, University College Dublin, Belfield, Dublin D04 V1W8, Ireland; ^3^ UCD Conway Institute of Biomolecular and Biomedical Research, University College Dublin, Belfield, Dublin D04 V1W8, Ireland; ^4^ Institute of Evolutionary Biology, School of Biological Sciences, University of Edinburgh, Edinburgh EH9 3FL, UK

**Keywords:** inbreeding, inbreeding depression, genomics, runs of homozygosity, racing, EFNA5

## Abstract

Small effective population sizes and active inbreeding can lead to inbreeding depression due to deleterious recessive mutations exposed in the homozygous state. The Thoroughbred racehorse has low levels of population genetic diversity, but the effects of genomic inbreeding in the population are unknown. Here, we quantified inbreeding based on runs of homozygosity (ROH) using 297 K SNP genotypes from 6128 horses born in Europe and Australia, of which 13.2% were unraced. We show that a 10% increase in inbreeding (*F*_ROH_) is associated with a 7% lower probability of ever racing. Moreover, a ROH-based genome-wide association study identified a haplotype on ECA14 which, in its homozygous state, is linked to a 32.1% lower predicted probability of ever racing, independent of *F*_ROH_. The haplotype overlaps a candidate gene, *EFNA5*, that is highly expressed in cartilage tissue, which when damaged is one of the most common causes of catastrophic musculoskeletal injury in racehorses. Genomics-informed breeding aiming to reduce inbreeding depression and avoid damaging haplotype carrier matings will improve population health and racehorse welfare.

## 1. Introduction

Thoroughbred horses are valuable domestic animals bred for competitive racing [1–3]. The small number of founders [4] and human-mediated selection that disproportionately favours the preservation of popular sire-lines [5] has led to an inbred population with a small effective population size [6,7]. Purposeful inbreeding is common, with breeders attempting to leverage beneficial genetic variants by choosing mates with shared ancestors such that multiple ancestors may be duplicated in a five-generation pedigree. A recent trend of increasing inbreeding has been observed in the population [5]. Inbreeding commonly leads to inbreeding depression, the reduced fitness of individuals due to deleterious recessive (or partially recessive) mutations exposed in the homozygous state [8,9]. In livestock, for every 1% increase in (pedigree-estimated) inbreeding, there is an estimated 0.13% decrease in the mean selected trait value [10].

Runs of homozygosity (ROH), long stretches of the genome identically inherited from both parents, can occur as the result of active inbreeding or other demographic processes that reduce effective population size [11]. The proportion of the genome covered by ROH affects production traits in livestock populations [12,13]. Generally, shorter ROH reflect distant inbreeding resulting from a common ancestor many generations back in the pedigree, whereas long ROH reflect a more recent common ancestor [14]. Mutations in long ROH are expected to be more harmful (deleterious) than those in short ROH since there have been fewer generations for purging to occur. In support of this, long ROH are associated with stronger inbreeding depression than short ROH in wild Soay sheep [15] and in humans, deleterious variants are enriched in long ROH [16]. However, in cattle deleterious variants are enriched in short and medium ROH, suggested to be due to hitchhiking with selected beneficial alleles [17].

In Thoroughbreds, a lower-than-expected mutational load has led to the hypothesis that existing selection processes are effective in purging deleterious mutations [7]. This may be facilitated by the unusually large census population size relative to the effective population size [5]; for instance, although racing is the breeding goal, a high proportion (35–50%) of foals born never race [18–22] and a small proportion enter the breeding population (less than 5% of males and 50% of females). While this selection may purge large-effect mutations it is expected that even at low frequencies, segregation of deleterious alleles within a population with a low effective population size will have consequences for some aspects of population viability [8] and inbreeding depression is likely to be persistent even with ongoing purging [15]. An analysis of pedigree-based inbreeding (Wright's inbreeding coefficient, *F*) in the Thoroughbred found a strong negative relationship between inbreeding and racing performance measures [23], which concluded that there is still considerable genetic load within the population.

To some degree, inbreeding is unavoidable in closed breeding populations [24]. Understanding how inbreeding may affect individual and population fitness is critical to improve breeding management and, for the Thoroughbred, may have both economic and welfare implications. For Thoroughbred breeders, the breeding goal is to produce viable foals that will have productive racing careers and establish their value by winning races. The number of races that an individual horse participates in and a horse's career duration are considered key indicators of animal health [25]. At a population level, it has been proposed that the success of the Thoroughbred industry may be measured by the proportion of horses born that commence racing [22].

Here, we used genome-wide SNP genotypes in a large cohort of Thoroughbred horses to examine the effects of genome-wide inbreeding on the probability of ever racing. Then, to identify regions of the genome containing large-effect loci, we performed a ROH-based genome-wide association analysis for the probability of racing in the population.

## 2. Methods

### (a) Phenotypes, genotype filtering and imputation

SNP genotypes, race region and year of birth were available for *n* = 8951 Thoroughbred horses. Race records (up to the end of the 2020 racing season) were retrieved for *n* = 6128 of these horses that were born in Europe (EUR) and Australia and New Zealand (ANZ) prior to and including 2015, and were therefore at least five years old. Among horses that race, the majority have their first start before they are five years old [22], and the median age of retirement from racing has been reported as five years old [19]. Horses were assigned as ‘raced’ (*n* = 3038, EUR, *n* = 2282 ANZ) if they had at least one start before five years old or ‘unraced’ (*n* = 606 EUR, *n* = 202 ANZ) if they had no recorded race start before five years old. Race records for the major race regions (Europe, Australia and North America) were used to partition samples into the two cohorts searching all regions including other than birth region. We cannot, however, rule out that horses categorized as ‘unraced’ may have raced in (minor) regions of the world that were not searched.

Two SNP genotyping platforms were used to genotype the animals; *n* = 4933 were genotyped on the Illumina Equine SNP70 BeadChip (Illumina, San Diego, CA) comprising approximately 70 000 SNPs (SNP70) and *n* = 4018 were genotyped on the Axiom Equine Genotyping Array (Axiom MNEC670) (Affymetrix, Santa Clara, CA) comprising approximately 670 000 SNPs (SNP670). All samples had a call rate greater than 95%. Samples genotyped on the SNP70 array were imputed up to 488 576 SNPs with BEAGLE v. 5.2 [26] using the samples genotyped on the SNP670 genotyping platform as a reference set. See electronic supplementary material, tables S1 and S2 for full details of the analysis cohorts.

### (b) Post-imputation filtering, quality control and population structure

After imputation, we discarded SNPs with a Beagle dosage *R*^2^ less than 0.8 to remove poorly imputed SNPs. We then filtered for SNPs with call rates greater than 0.99, minor allele frequency greater than or equal to 0.01 and retained only SNPs located on autosomes, leaving a final dataset with 296 691 SNPs. Based on this dataset, we conducted a principal component analysis in PLINK v1.90b [27], which showed only minor population stratification between EUR and ANZ horses in our dataset (electronic supplementary material, figure S1).

### (c) Quantifying runs of homozygosity and inbreeding (*F*_ROH_)

We called ROH with a minimum length of 300 kb using -homozyg in PLINK v1.90b [27] with the following parameters: -homozyg-window-snp 30 -homozyg-snp 30 -homozyg-kb 300 -homozyg-gap 150 -homozyg-density 100 -homozyg-window-missing 1 -homozyg-window-het 1. We chose 300 kb as the minimum ROH length as we were interested in evaluating fitness effects of both shorter and longer ROH. Moreover, based on the SNP density and horse genome size, we expect around 40 SNPs in a 300 kb stretch of ROH, which should be sufficient to reliably call short ROH.

Inbreeding coefficients (*F*_ROH_) were calculated by summing the total length of ROH for each individual and dividing by the autosomal genome length of 2281 Mb [28,29]. Shorter ROH with most-recent common ancestors from further back in the pedigree might differ in their fitness effects compared to long ROH [15–17]. We therefore also calculated an inbreeding coefficient for ROH less than 5 Mb (*F*_ROH_short_) and for ROH ≥ 5 Mb (*F*_ROH_long_). While the cutoff is semi-arbitrary, ROH of length 5 Mb are expected to have a common ancestor haplotype approximately 10 generations ago, calculated as $\frac{100}{2\times g}$ with *g* being the number of generations [30], assuming a uniform recombination map such that 1 Mb is equivalent to 1 cM. The effects of *F*_ROH_long_ and *F*_ROH_short_ can therefore be broadly interpreted as effects of more recent versus older inbreeding, or more precisely, of younger versus older haplotypes.

### (d) Modelling inbreeding effects on fitness to race

We modelled the effects of inbreeding on racing using general linear mixed models in lme4 [31]. A horse was assigned 0 if it had never raced and 1 if it had raced, and we therefore used a binomial error distribution with logit link with the following model structure:

$$\begin{array}{rl} \mathrm{Pr}(raced_{i}=1) & =logit^{-1}(\beta_{0}+\;F_{ROH_{i}}\beta_{1}+\;region_{i}\beta_{2}+\;sex_{i}\beta_{3}\;+\;\alpha_{k}^{birth\;year})\\ & \;\alpha_{k}^{birth\;year}∼N(0,\sigma_{birth\;year}^{2}),\;\mathrm{for}\;\;k=1,\ldots ,38. \end{array}$$

The probability of having raced Pr(raced_i_ = 1) was modelled with an intercept $\beta_{0}$ and fixed effects for individual inbreeding *F*_ROH_, *region* (ANZ and EUR) and *sex* (F, M). We also fitted birth year as a random effect in the model. The sample size was *n* = 6128, with 2484 and 3644 horses from ANZ and EUR, respectively. To disentangle effects of short and long ROH, we re-fitted the model and replaced *F*_ROH_ with two predictors *F*_ROH_short_ and *F*_ROH_long_.

### (e) Mapping loci underpinning inbreeding depression in fitness to race

To detect potential large-effect loci involved in inbreeding depression and to distinguish these from additive SNP effects, we performed a ROH-based genome-wide association study (GWAS) [13,15]. At every SNP location, we fitted a binomial mixed model with logit link using lme4 with the following structure:

$$\begin{array}{rl} \mathrm{Pr}(raced_{i}=1) & =logit^{-1}(\beta_{0}+\;SNP_{ROH_{alleleA}}{\;}_{i}\beta_{1}\\ & +\;SNP_{ROH_{alleleB}}{\;}_{i}\beta_{2}+\;SNP_{ADD_{i}}\beta_{3}\\ & +\;F_{ROH_{mod}}{\;}_{i}\beta_{4}+\;region_{i}\beta_{5}+\;sex_{i}\beta_{6}+\beta_{7-17}PC_{1-10}\\ & +\;\alpha_{j}^{birth\;year}\alpha_{k}^{birth\;year}\;\;∼\;N(0,\sigma_{birth\;year}^{2}),\;\mathrm{for}\;k=1,\ldots ,38. \end{array}$$

The predictors of interest are $SNP_{ROH_{alleleA}}{\;}_{i}$ and $SNP_{ROH_{alleleB}}{\;}_{i}$, which are binary predictors quantifying whether an individual had a ROH overlapping a given SNP position with allele A as the homozygous genotype $\left(SNP_{ROH_{alleleA}}{\;}_{i}=1\right)$ or with allele B as the homozygous genotype $\left(SNP_{ROH_{alleleB}}{\;}_{i}=1\right)$. When the SNP was not in a ROH it was coded as 0. These predictors indicate whether ROH have an effect on the probability of racing at a given position in the genome. The reason for fitting two ROH predictors is that a large-effect deleterious allele is likely to occur only on certain haplotypic backgrounds. To ensure that a ROH effect is not simply an additive effect, we also fitted a predictor with the SNP genotype coded as 0,1,2 for homozygous, heterozygous and homozygous for the alternative allele. $F_{ROH_{mod}}$ is the individual inbreeding coefficient calculated from all autosomes except for the chromosome containing the focal SNP. This ensures that the estimated effects are local ROH effects separate to the genome-wide inbreeding level. As before, we fitted region and sex as further fixed effects as well as 10 principal components based on the variance-standardized additive relationship matrix to account for relatedness within the sample. Lastly, we again included year of birth as a random effect. For each model, we extracted the model estimates for both ROH effects and their *p*-values calculated using Wald Z tests. We determined a genome-wide significance threshold by calculating the effective number of tests when accounting for linkage disequilibrium using ‘simpleM’ [32]. This led to an estimated 90 900 independent tests, which we doubled (as two tests per model were performed), before using this value for a Bonferroni correction of *p*-values, leading to a genome-wide significance threshold of *p* < 2.75 × 10^−7^.

## 3. Results

### (a) Patterns of inbreeding in the genome and across time

First, we evaluated ROH using 297 K SNP genotypes generated for *n* = 8951 Thoroughbred horses born in EUR and ANZ between 1971 and 2020. On average, individual animals had 415 (range 229–994) ROH segments greater than 300 kb with a mean length of 1.5 Mb (the longest ROH spanned 67 Mb) that covered approximately 28% of each genome (mean *F*_ROH_ = 0.28; range 0.18–0.40) (electronic supplementary material, figure S2). Out of these, on average 24 (range 0–49) were long ROH greater than 5 Mb and 391 (range 228–994) were short ROH less than 5 Mb, covering 8.7% and 19.5% of the genome, respectively. The frequency of ROH varied across the genome, including hotspots where up to 90% of horses have ROH and coldspots where ROH were rare (electronic supplementary material, figure S3), in line with high variation observed in other species [15,33]. Consistent with our previous report [5] we observed an increase in *F*_ROH_ in both EUR and ANZ over time (electronic supplementary material, figure S4).

### (b) Inbreeding effects on racing

**Figure 1.**
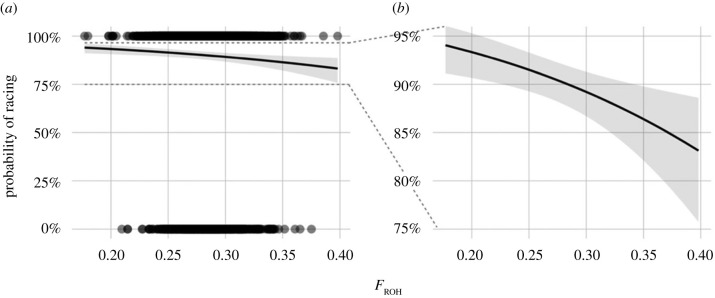
Predicted probability (and 95% confidence intervals) of racing for different inbreeding coefficients (*F*_ROH_). (*a*) Predictions shown alongside raw data (horses that have raced at 100% and those that have not raced at 0%). (*b*) Close-up of the relevant plotting area shown in (*a*).

To test the hypothesis that inbreeding influences failure to race, we modelled the effects of inbreeding (*F*_ROH_) on the probability of racing among *n* = 6128 horses using general linear mixed models. The log-odds of racing decreased significantly (*p* = 0.0009) with increasing *F*_ROH_ (log-odds ratio (log(OR) [95% confidence interval (CI) = −5.75 [−9.15, −2.36], electronic supplementary material, table S3), corresponding to a predicted decrease in the odds of racing by 44% for a 10% increase in *F*_ROH_. It is also possible to translate these effects into predicted probabilities of racing (figure 1). According to the model, a horse with the highest observed inbreeding coefficient (*F*_ROH_ = 0.40) had roughly a 13% lower probability of ever racing than a horse with the lowest observed inbreeding coefficient (*F*_ROH_ = 0.18). Horses that were 10% more inbred than average had a 7% lower probability of ever racing, while horses with a *F*_ROH_ 10% lower than the mean had a 4% higher probability of racing (see electronic supplementary material, table S4 for numeric predictions). To test for differences in inbreeding depression between EUR and ANZ horses, we also fitted a model with an interaction between *F*_ROH_ and region (EUR and ANZ), but the slopes were very similar and the interaction was not significant (electronic supplementary material, figure S5 and table S5). In addition to modelling inbreeding effects on whether a horse had raced at all, we also fitted a model to test whether *F*_ROH_ is linked to a lower number of races among those horses that did race, but here *F*_ROH_ was not statistically significant (electronic supplementary material, table S6).

### (c) Inbreeding effects on racing by runs of homozygosity length

**Figure 2.**
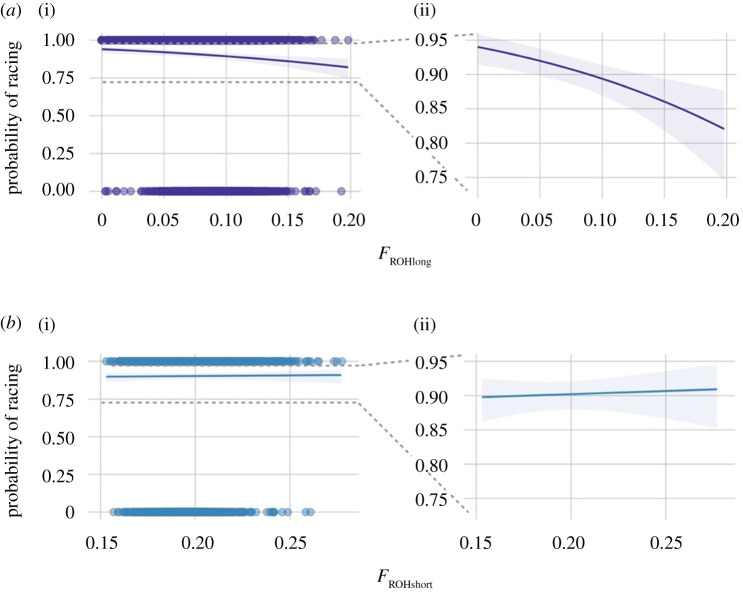
Predicted probability (and 95% confidence intervals) of racing for different inbreeding coefficients *F*_ROH_ based on (*a*) long ROH greater than 5 Mb and (*b*) short ROH less than 5 Mb. (*a*)(i) and (*b*)(i) show predictions alongside raw data (horses that have raced at 100% and those that have not raced at 0%); (*a*)(ii) and (*b*)(ii) zoom closer into the relevant plotting area. (Online version in colour.)

Long ROH had a strong negative effect on the probability of racing, while short ROH had no effect (figure 2; electronic supplementary material, table S7), indicating that recent inbreeding rather than historic inbreeding is the cause of inbreeding depression for this trait in the population. The predicted log-odds of racing decreased with increasing *F*_ROH_long_ (log(OR) [95% CI] = −6.57 [−10.04, −3.11]) corresponding to a predicted decrease in the odds of racing by 48% for a 10% increase in *F*_ROH_long_.

### (d) Identification of a large-effect haplotype

**Figure 3.**
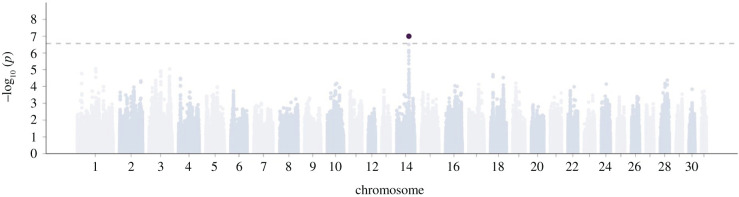
GWAS of ROH effects on the probability of ever racing. The Manhattan plot shows *p*-values for ROH effects on the probability of racing at every SNP location across the genome. Per SNP, two effects of ROH were tested to differentiate between ROH containing the minor allele and ROH containing the major allele. The hit on chromosome 14 corresponds to a negative effect of ROH on the probability of racing. (Online version in colour.)

The GWAS uncovered a single significant hit (*p* = 9.09 × 10^−8^) for a ROH overlapping the minor allele A at SNP rs1144708552 at position 63883487 bp on ECA14 (log(OR) [95% CI] = −1.82 [−2.48, −1.15]; figure 3; electronic supplementary material, figure S6 and table S8). While only 64 individuals (1%) were homozygous for the A allele and had overlapping ROH (ROH + AA), these horses had a 32% lower predicted probability of ever racing (electronic supplementary material, table S8) when keeping other factors such as *F*_ROH_ constant. The 64 ROH + AA horses were the progeny of 40 sires. 31.25% of ROH + AA horses were unraced, compared to the sample average 13.1%. Furthermore, ROH + AA horses that did race had on average 13.3 races, compared to the sample average of 16.3 races, suggesting that while some ROH + AA horses may race, they are less durable. Notably, genome-wide inbreeding (*F*_ROH_) also showed a strong effect on the probability of racing in the same model (log(OR) [95% CI] = −6.39 [−9.88, −2.90]; electronic supplementary material, table S9). Consequently, inbreeding depression in the Thoroughbred is largely due to the genome-wide effects of many recessive deleterious mutations in addition to a larger effect recessive mutation expected to be in proximity to SNP rs1144708552.

Using phased genotype data, we screened haplotypes surrounding the rs1144708552 SNP. Specifically, we focussed on haplotypes of length 1 Mb, including all SNPs occurring up to 500 kb on each side of rs1144708552. Among ROH + AA horses, 78.1% were homozygous for an identical haplotype (electronic supplementary material, table S10), which we refer to as THR14. All but one ROH + AA horse carried at least one exact copy of THR14, and where haplotypes differed from THR14, this was on average by only 2 out of 84 base positions that make up the haplotype. THR14 was significantly (*p* = 0.006) overrepresented among breeding stallions (*n* = 130), where it had a frequency of 15.4% compared to 10.1% in the overall sample.

### (e) Candidate genes for survival/viability

Since most (approx. 80%) of the ROH in the identified region extend across greater than 2.8 Mb we searched a 3 Mb region surrounding the rs1144708552 SNP (±1.5 Mb) for genes with biological functions that may impact on survival or viability of a horse. The most compelling candidate gene among the three protein-coding genes (*FER*, *FBXL17* and *EFNA5*) in the region was *EFNA5* (ECA14: 63365481–63631761 bp), which was also the closest (approx. 250 kb) gene to the rs1144708552 SNP (electronic supplementary material, figure S7).

## 4. Discussion

Inbreeding in livestock populations is difficult to avoid due to the often closed nature of the gene pool. Nonetheless, limiting inbreeding is likely to reduce the impact of undesirable inherited features. Until now, the functional or clinical consequences of inbreeding in the Thoroughbred population were not known. Here we show, using SNP genotypes for a large cohort of Thoroughbred horses in two of the major global breeding regions, that inbreeding depression is a genome-wide phenomenon significantly impacting on the viability of a horse to ever race. Given the current pedigree structure of Thoroughbreds, avoiding breeding close relatives is challenging and the increasing levels of inbreeding in the population indicates that existing strategies to mitigate inbreeding may be inadequate. Some breeders consider that the duplication of influential ancestral horses in a pedigree may be advantageous and there are numerous examples of close pedigree inbreeding that result in successful racehorses. Indeed, here we show that inbreeding in the distant pedigree, measured as *F*_ROH_short_, is not disadvantageous to the breeding goal. This observation is in agreement with an analysis of pedigree-based inbreeding in the Australian Thoroughbred population that suggested that the ancestral history coefficient of inbreeding, the number of times an allele has been identical by descent in an individual's pedigree, has a positive association with racing performance and probably captures the effects of positive selection for favourable exercise-relevant traits over many generations [23]. However, more recently shared common ancestors, indicated by *F*_ROH_long_, have a considerable negative impact on the viability of a horse for racing and contribute to wastage in the population. Although not quantified here, it is likely that these long ROH contain a higher proportion of rare, deleterious alleles, which cumulatively cause the inbreeding depression observed.

Large-effect mutations are expected to be more easily purged by selection [8]. However, here as well as describing the genome-wide architecture of inbreeding, we have identified a relatively common (10.1%) haplotype with an auxiliary effect on probability to race. This haplotype did not overlap any known locus under selection for Thoroughbreds [5,34–36]. Nonetheless, there was a higher than expected frequency of the THR14 haplotype among stallions, which occurs in one in six breeding stallions in our sample. Estimating from the frequency of the haplotype in our sample (10.1%), among the 8542 foals registered in Ireland in 2020, 87 are expected to be homozygous for the THR14 haplotype with 1551 carriers.

*EFNA5* (ECA14: 63 365 481–63 631 761 bp), which encodes the ephrin ligand, ephrin-A5, is a compelling candidate gene for the negative effect of the THR14 haplotype on racing. Ephrin A5 is a member of the ephrin family of ligands and receptors that are broadly expressed during tissue development and repair [37–42]. Ephrin A5 is involved in neonatal muscle development and regeneration, regulates cardiomyocytes and is associated with growth traits and digital cushion thickness (a strong predictor of lameness) in cattle [42–46]. Ephrin ligand–receptor signalling also has a well characterised role in skeletal development and bone function [47–49]. Generally, ephrin A member ligands bind to ephrin A receptors; however, ephrin A5 is known to bind to the ephrin B2 receptor [50], which is encoded by a gene involved in osteogenic control and hip fracture in humans [51,52]. The related ephrin B1 protein is required for the fracture repair process [53]. Ephrin A5 has been proposed as a negative regulator of osteogenic differentiation [54] and is highly expressed in cartilage [55] where it participates in tissue growth and regeneration [56]. Although articular cartilage is a highly resilient tissue, it has a poor capacity for regeneration and articular cartilage damage in horses is one of the most common causes of catastrophic musculoskeletal injury [57]. Pre-existing degenerative articular cartilage and bone lesions have been commonly identified in euthanized horses post-injury [58] resulting from cumulative damage to the tissues from repetitive strain during exercise [59,60]. In this regard, the THR14 haplotype overlaps a large suggestive GWAS peak on ECA14 (14: 55530859–70816518) for osteochondrosis in the Belgian Warmblood breed [61]. Osteochondrosis is one of the most common skeletal diseases in the horse affecting cartilage and bone, causing inflammation of joints, pain, lameness and a decrease in athletic performance.

There are many reasons that a foal may not become a viable racehorse [18–20]. However, musculoskeletal fractures are the most common cause of death at all stages of development, training and racing [19], and are a major concern for racehorse welfare. The known biological functions of *EFNA5* and the haplotype association with the probability of racing that we report here lead to the hypothesis that it may play a role in musculoskeletal injury risk; however, this must be tested in a population of horses with well-defined phenotypes. While the nature of the hypothesized causative mutation on THR14 is not known, we provide here strong evidence for the presence of a mutation with a negative effect on racing located within this haplotype that has not previously been described.

In summary, the introduction of genome-enabled breeding strategies to avoid the production of long homozygous stretches in the genome and homozygosity of the THR14 haplotype could improve economic returns for breeders and positively impact animal welfare. Therefore, industry-guided monitoring of genome-wide inbreeding over time will be important to maintain sustainable populations of horses, with particular attention focused on higher resolution information that can be obtained for deleterious haplotypes such as THR14.

## Data Availability

The raw data analysed in this study are subject to the following licences/restrictions: the phenotype and genotype data analysed in the present study are the property of Plusvital Ltd and subject to a confidentiality agreement with the animal owners. Requests to access the datasets should be directed to E.W.H. *F_ROH_* measures and phenotypes are available from the Dryad Digital Repository: https://doi.org/10.5061/dryad.rn8pk0pcr [62]. Electronic supplementary material is available online [63].

## References

[1] Mcmanus P, Albrecht G, Graham R. 2012 The global horseracing industry: social, economic, environmental and ethical perspectives, 1st edn. London, UK: Routledge.

[2] Cassidy R. 2002 The sport of kings: kinship, class and Thoroughbred breeding in newmarket. Cambridge, UK: Cambridge University Press.

[3] Willett P. 1970 The Thoroughbred. New York: NY: G.P. Putnam's Sons.

[4] Cunningham EP, Dooley JJ, Splan RK, Bradley DG. 2001 Microsatellite diversity, pedigree relatedness and the contributions of founder lineages to Thoroughbred horses. Anim. Genet. **32**, 360-364. (10.1046/j.1365-2052.2001.00785.x) 11736806

[5] Mcgivney BA, Han H, Corduff LR, Katz LM, Tozaki T, Machugh DE, Hill EW. 2020 Genomic inbreeding trends, influential sire lines and selection in the global Thoroughbred horse population. Sci. Rep. **10**, 466. (10.1038/s41598-019-57389-5) 31949252 PMC6965197

[6] Petersen JL et al. 2013 Genetic diversity in the modern horse illustrated from genome-wide SNP data. PLoS ONE **8**, e54997. (10.1371/journal.pone.0054997) 23383025 PMC3559798

[7] Orlando L, Librado P. 2019 Origin and evolution of deleterious mutations in horses. Genes (Basel) **10**. (10.3390/genes10090649) PMC6769756 31466279

[8] Charlesworth D, Willis JH. 2009 The genetics of inbreeding depression. Nat. Rev. Genet. **10**, 783-796. (10.1038/nrg2664) 19834483

[9] Hedrick PW, Garcia-Dorado A. 2016 Understanding inbreeding depression, purging, and genetic rescue. Trends Ecol. Evol. **31**, 940-952. (10.1016/j.tree.2016.09.005) 27743611

[10] Doekes HP, Bijma P, Windig JJ. 2021 How depressing is inbreeding? A meta-analysis of 30 years of research on the effects of inbreeding in livestock. Genes (Basel) **12**. (10.3390/genes12060926) PMC8234567 34207101

[11] Ceballos FC, Joshi PK, Clark DW, Ramsay M, Wilson JF. 2018 Runs of homozygosity: windows into population history and trait architecture. Nat. Rev. Genet. **19**, 220-234. (10.1038/nrg.2017.109) 29335644

[12] Ferencakovic M, Solkner J, Kaps M, Curik I. 2017 Genome-wide mapping and estimation of inbreeding depression of semen quality traits in a cattle population. J. Dairy Sci. **100**, 4721-4730. (10.3168/jds.2016-12164) 28434751

[13] Pryce JE, Haile-Mariam M, Goddard ME, Hayes BJ. 2014 Identification of genomic regions associated with inbreeding depression in Holstein and Jersey dairy cattle. Genet. Sel. Evol. **46**, 71. (10.1186/s12711-014-0071-7) 25407532 PMC4234836

[14] Sumreddee P, Hay EH, Toghiani S, Roberts A, Aggrey SE, Rekaya R. 2021 Grid search approach to discriminate between old and recent inbreeding using phenotypic, pedigree and genomic information. BMC Genomics **22**, 538. (10.1186/s12864-021-07872-z) 34256689 PMC8278650

[15] Stoffel MA, Johnston SE, Pilkington JG, Pemberton JM. 2021 Mutation load decreases with haplotype age in wild Soay sheep. Evol. Lett. **5**, 187-195. (10.1002/evl3.229) 34136268 PMC8190445

[16] Szpiech ZA, Xu J, Pemberton TJ, Peng W, Zöllner S, Rosenberg NA, Li JZ. 2013 Long runs of homozygosity are enriched for deleterious variation. Am. J. Hum. Genet. **93**, 90-102. (10.1016/j.ajhg.2013.05.003) 23746547 PMC3710769

[17] Zhang Q, Guldbrandtsen B, Bosse M, Lund MS, Sahana G. 2015 Runs of homozygosity and distribution of functional variants in the cattle genome. BMC Genomics **16**, 542. (10.1186/s12864-015-1715-x) 26198692 PMC4508970

[18] Flash ML, Wong ASM, Stevenson MA, Gilkerson JR. 2020 Barriers to entering race training before 4 years of age for Thoroughbred horses born in the 2014 Australian foal crop. PLoS ONE **15**, e0237003. (10.1371/journal.pone.0237003) 32756576 PMC7406052

[19] Flash ML, Renwick M, Gilkerson JR, Stevenson MA. 2020 Descriptive analysis of Thoroughbred horses born in Victoria, Australia, in 2010; barriers to entering training and outcomes on exiting training and racing. PLoS ONE **15**, e0241273. (10.1371/journal.pone.0241273) 33112903 PMC7592779

[20] Galvin N, Corley K. 2010 Causes of disease and death from birth to 12 months of age in the Thoroughbred horse in Ireland. Ir. Vet. J. **63**, 37-43. (10.1186/2046-0481-63-1-37) 21851741 PMC3113843

[21] Jeffcott LB, Rossdale PD, Freestone J, Frank CJ, Towers-Clark PF. 1982 An assessment of wastage in Thoroughbred racing from conception to 4 years of age. Equine Vet. J. **14**, 185-198. (10.1111/j.2042-3306.1982.tb02389.x) 7106081

[22] Flash ML, Crabb HK, Hitchens PL, Firestone SM, Stevenson MA, Gilkerson JR. 2022 Participation of Victorian Thoroughbreds in the racing industry: a whole-of-population benchmark. Aust. Vet. J. **100**, 40-47. (10.1111/avj.13124) 34595748

[23] Todd ET, Ho SYW, Thomson PC, Ang RA, Velie BD, Hamilton NA. 2018 Founder-specific inbreeding depression affects racing performance in Thoroughbred horses. Sci. Rep. **8**, 6167. (10.1038/s41598-018-24663-x) 29670190 PMC5906619

[24] Leroy G. 2014 Inbreeding depression in livestock species: review and meta-analysis. Anim. Genet. **45**, 618-628. (10.1111/age.12178) 24975026

[25] Flash ML, Crabb HK, Hitchens PL, Firestone SM, Stevenson MA, Gilkerson JR. 2022 Factors associated with racing performance and career duration for Victorian-born Thoroughbreds. Aust. Vet. J. **100**, 48-55. (10.1111/avj.13128) 34651302

[26] Browning BL, Zhou Y, Browning SR. 2018 A one-penny imputed genome from next-generation reference panels. Am. J. Hum. Genet. **103**, 338-348. (10.1016/j.ajhg.2018.07.015) 30100085 PMC6128308

[27] Purcell S et al. 2007 PLINK: a tool set for whole-genome association and population-based linkage analyses. Am. J. Hum. Genet. **81**, 559-575. (10.1086/519795) 17701901 PMC1950838

[28] Wade CM et al. 2009 Genome sequence, comparative analysis, and population genetics of the domestic horse. Science **326**, 865-867. (10.1126/science.1178158) 19892987 PMC3785132

[29] Mcquillan R et al. 2008 Runs of homozygosity in European populations. Am. J. Hum. Genet. **83**, 359-372. (10.1016/j.ajhg.2008.08.007) 18760389 PMC2556426

[30] Thompson EA. 2013 Identity by descent: variation in meiosis, across genomes, and in populations. Genetics **194**, 301-326. (10.1534/genetics.112.148825) 23733848 PMC3664843

[31] Bates DM, Mächler M, Bolker B, Walker S. 2015 Fitting linear mixed-effects models using lme4. J. Stat. Softw. **67**, 1-48. (10.18637/jss.v067.i01)

[32] Gao X, Starmer J, Martin ER. 2008 A multiple testing correction method for genetic association studies using correlated single nucleotide polymorphisms. Genet. Epidemiol. **32**, 361-369. (10.1002/gepi.20310) 18271029

[33] Kardos M et al. 2018 Genomic consequences of intensive inbreeding in an isolated wolf population. Nat. Ecol. Evol. **2**, 124-131. (10.1038/s41559-017-0375-4) 29158554

[34] Petersen JL et al. 2013 Genome-wide analysis reveals selection for important traits in domestic horse breeds. PLoS Genet. **9**, e1003211. (10.1371/journal.pgen.1003211) 23349635 PMC3547851

[35] Han H, Mcgivney BA, Farries G, Katz LM, Machugh DE, Randhawa IAS, Hill EW. 2020 Selection in Australian Thoroughbred horses acts on a locus associated with early two-year old speed. PLoS ONE **15**, e0227212. (10.1371/journal.pone.0227212) 32049967 PMC7015314

[36] Fawcett JA, Sato F, Sakamoto T, Iwasaki WM, Tozaki T, Innan H. 2019 Genome-wide SNP analysis of Japanese Thoroughbred racehorses. PLoS ONE **14**, e0218407. (10.1371/journal.pone.0218407) 31339891 PMC6655603

[37] Edwards CM, Mundy GR. 2008 Eph receptors and ephrin signaling pathways: a role in bone homeostasis. Int. J. Med. Sci. **5**, 263-272. (10.7150/ijms.5.263) 18797510 PMC2536716

[38] Nguyen TM, Arthur A, Paton S, Hemming S, Panagopoulos R, Codrington J, Walkley CR, Zannettino ACW, Gronthos S. 2016 Loss of ephrinB1 in.osteogenic progenitor cells impedes endochondral ossification and compromises bone strength integrity during skeletal development. Bone **93**, 12-21. (10.1016/j.bone.2016.09.009) 27622886

[39] Stark DA, Karvas RM, Siegel AL, Cornelison DD. 2011 Eph/ephrin interactions modulate muscle satellite cell motility and patterning. Development **138**, 5279-5289. (10.1242/dev.068411) 22071104 PMC3222207

[40] D'Souza D, Patel K. 1999 Involvement of long- and short-range signalling during early tendon development. Anat Embryol (Berl) **200**, 367-375. (10.1007/s004290050286) 10460474

[41] Compagni A, Logan M, Klein R, Adams RH. 2003 Control of skeletal patterning by ephrinB1-EphB interactions. Dev. Cell **5**, 217-230. (10.1016/S1534-5807(03)00198-9) 12919674

[42] Alonso-Martin S, Rochat A, Mademtzoglou D, Morais J, De Reynies A, Auradé F, Chang THT, Zammit PS, Relaix F. 2016 Gene expression profiling of muscle stem cells identifies novel regulators of postnatal myogenesis. Front. Cell Dev. Biol. **4**, 58. (10.3389/fcell.2016.00058) 27446912 PMC4914952

[43] Gu JM et al. 2016 An NF-kappaB–ephrinA5-dependent communication between NG2^+^ interstitial cells and myoblasts promotes muscle growth in neonates. Dev. Cell **36**, 215-224. (10.1016/j.devcel.2015.12.018) 26777211 PMC4732710

[44] Li YY, Mi Z, Feng Y, Mctiernan CF, Zhou R, Robbins PD, Watkins SC, Feldman AM. 2001 Differential effects of overexpression of two forms of ephrin-A5 on neonatal rat cardiomyocytes. Am. J. Physiol. Heart Circ. Physiol. **281**, H2738-H2746. (10.1152/ajpheart.2001.281.6.H2738) 11709443

[45] An B et al. 2020 Multiple association analysis of loci and candidate genes that regulate body size at three growth stages in Simmental beef cattle. BMC Genet. **21**, 32. (10.1186/s12863-020-0837-6) 32171250 PMC7071762

[46] Stambuk CR, Staiger EA, Heins BJ, Huson HJ. 2020 Exploring physiological and genetic variation of digital cushion thickness in Holstein and Jersey cows and bulls. J. Dairy Sci. **103**, 9177-9194. (10.3168/jds.2020-18290) 32713698

[47] Lindsey RC, Rundle CH, Mohan S. 2018 Role of IGF1 and EFN-EPH signaling in skeletal metabolism. J. Mol. Endocrinol. **61**, T87-T102. (10.1530/JME-17-0284) 29581239 PMC5966337

[48] Matsuo K, Otaki N. 2012 Bone cell interactions through Eph/ephrin: bone modeling, remodeling and associated diseases. Cell Adh. Migr. **6**, 148-156. (10.4161/cam.20888) 22660185 PMC3499314

[49] Arthur A, Gronthos S. 2021 Eph-ephrin signaling mediates cross-talk within the bone microenvironment. Front. Cell Dev. Biol. **9**, 598612. (10.3389/fcell.2021.598612) 33634116 PMC7902060

[50] Himanen JP et al. 2004 Repelling class discrimination: ephrin-A5 binds to and activates EphB2 receptor signaling. Nat. Neurosci. **7**, 501-509. (10.1038/nn1237) 15107857

[51] Ruan Z, Zhu Y, Lin Z, Long H, Zhao R, Sun B, Cheng L, Zhao S. 2020 Association between rs12742784 polymorphism and hip fracture, bone mineral density, and EPHB2 mRNA expression levels in elderly Chinese women. Climacteric **23**, 93-98. (10.1080/13697137.2019.1640195) 31352841

[52] Nielson CM et al. 2016 Novel genetic variants associated with increased vertebral volumetric BMD, reduced vertebral fracture risk, and increased expression of SLC1A3 and EPHB2. J. Bone Miner. Res. **31**, 2085-2097. (10.1002/jbmr.2913) 27476799 PMC5477772

[53] Arthur A, Paton S, Zannettino ACW, Gronthos S. 2020 Conditional knockout of ephrinB1 in osteogenic progenitors delays the process of endochondral ossification during fracture repair. Bone **132**, 115189. (10.1016/j.bone.2019.115189) 31863961

[54] Yamada T et al. 2013 After repeated division, bone marrow stromal cells express inhibitory factors with osteogenic capabilities, and EphA5 is a primary candidate. Bone **57**, 343-354. (10.1016/j.bone.2013.08.028) 24029132

[55] Kalinski T, Ropke A, Sel S, Kouznetsova I, Ropke M, Roessner A. 2009 Down-regulation of ephrin-A5, a gene product of normal cartilage, in chondrosarcoma. Hum. Pathol. **40**, 1679-1685. (10.1016/j.humpath.2009.03.024) 19695673

[56] Guan M, Pan D, Zhang M, Leng X, Yao B. 2021 Deer antler extract potentially facilitates xiphoid cartilage growth and regeneration and prevents inflammatory susceptibility by regulating multiple functional genes. J. Orthop. Surg. Res. **16**, 208. (10.1186/s13018-021-02350-4) 33752715 PMC7983396

[57] Desjardins MR, Hurtig MB. 1990 Cartilage healing: a review with emphasis on the equine model. Can. Vet. J. **31**, 565-572. 17423644 PMC1480835

[58] Janes JG, Kennedy LA, Garrett KS, Engiles JB. 2017 Common lesions of the distal end of the third metacarpal/metatarsal bone in racehorse catastrophic breakdown injuries. J. Vet. Diagn. Invest. **29**, 431-436. (10.1177/1040638717717948) 28681688

[59] Martig S, Chen W, Lee PV, Whitton RC. 2014 Bone fatigue and its implications for injuries in racehorses. Equine Vet. J. **46**, 408-415. (10.1111/evj.12241) 24528139

[60] Turley SM, Thambyah A, Riggs CM, Firth EC, Broom ND. 2014 Microstructural changes in cartilage and bone related to repetitive overloading in an equine athlete model. J. Anat. **224**, 647-658. (10.1111/joa.12177) 24689513 PMC4025892

[61] Drabbe A et al. 2022 Genome-wide association analyses of osteochondrosis in belgian warmbloods reveal candidate genes associated with chondrocyte development. J. Equine Vet. Sci. **111**, 103870. (10.1016/j.jevs.2022.103870) 35074400

[62] Hill E et al. 2022 Thoroughbred horse inbreeding measures and racing phenotypes. Dryad Digital Repository. (10.5061/dryad.rn8pk0pcr)

[63] Hill EW, Stoffel MA, McGivney BA, MacHugh DE, Pemberton JM. 2022 Inbreeding depression and the probability of racing in the Thoroughbred horse. Figshare. (10.6084/m9.figshare.c.6035793) PMC9240673 35765835

